# GAVI supply chain strategy people and practice evidence review

**DOI:** 10.1186/2052-3211-7-S1-P6

**Published:** 2014-12-17

**Authors:** Pamela Steele

**Affiliations:** 1 Pamela Steele Associates (PSA) Ltd, Oxford, UK

## Background

It is estimated that in some cases up to 50% of vaccines are wasted by not being administered, where these supply chain inefficiencies may be contributing to the deaths of 1.5 million children each year from vaccine-preventable diseases. The GAVI Alliance partners and Secretariat, WHO, UNICEF, and the Bill & Melinda Gates Foundation are currently designing a supply chain strategy to increase investment, coordinate global activities, and ensure more children receive the vaccines they need.

## Method

This study adopted a systematic review of 47 documents using three techniques: bibliographic online searches using keywords, use of websites of international organizations that support, fund or monitor issues related to health supply chains, and finally, a grey literature search used to unearth further information by examining and following up sources from different websites. A working group consisting of health supply chain specialists provided the author with expert advice and guidance on both the GAVI strategy and sources of literature.

## Results

Although many significant results have been achieved and important targets are on their way to be reached, there is recognition of the existence of multiple challenges, which are representative of the immunization systems in developing countries. They have been identified as: ministries of health leadership and staff are not empowered to make critical decisions, the supply chain management organization is inadequately designed to face the increasing complexity, lack of qualified staff performing supply chain functions with limited access to adequate training, absence of a proper incentive and performance management system, poor logistics practices resulting in wastage and stock-outs.

## Discussion

A clear direction arises from this study, which combines Human Resource for Health (HRH) practices and supply chain management capabilities. The issues discussed in each hypothesis are in reality interconnected in a complex web, which HRH theory goes some way to explain.

While human resources issues in immunization supply chains need to be considered in conjunction with other critical supply chain areas including: system design, data management, cold chain equipment, transport and distribution.

## Lessons learned

This report has revealed, using a snapshot of the existing literature, that there is a paucity of research on human resources for global health supply chains in developing countries.

